# Fabrication of Metal Matrix Composite by Laser Metal Deposition—A New Process Approach by Direct Dry Injection of Nanopowders

**DOI:** 10.3390/ma12213584

**Published:** 2019-10-31

**Authors:** Briac Lanfant, Florian Bär, Antaryami Mohanta, Marc Leparoux

**Affiliations:** Empa–Swiss Federal Laboratories for Materials Science and Technology, Laboratory for Advanced Materials Processing, 3602 Thun, Switzerland; briac.lanfant@empa.ch (B.L.); florian.baer@empa.ch (F.B.); antaryami.mohanta@empa.ch (A.M.)

**Keywords:** laser metal deposition, direct nanopowder injection, metal matrix composite, lamellar microstructure, titanium aluminide, solid solution, hardness

## Abstract

Laser Metal Deposition (LMD) offers new perspectives for the fabrication of metal matrix nanocomposites (MMnCs). Current methods to produce MMnCs by LMD systematically involve the premixing of the nanopowders and the micropowders or require in-situ strategies, thereby restricting the possibilities to adjust the nature, content and location of the nano-reinforcement during printing. The objective of this study is to overcome such restrictions and propose a new process approach by direct injection of nanoparticles into a metallic matrix. Alumina (n-Al_2_O_3_) nanoparticles were introduced into a titanium matrix by using two different direct dry injection modes in order to locally increase the hardness. Energy dispersive X-ray spectroscopy (EDS) analyses validate the successful incorporation of the n-Al_2_O_3_ at chosen locations. Optical and high resolution transmission electron microscopic (HR-TEM) observations as well as X-ray diffraction (XRD) analyses indicate that n-Al_2_O_3_ powders are partly or totally dissolved into the Ti melted pool leading to the in-situ formation of a composite consisting of fine α_2_ lamellar microstructure within a Ti matrix and a solid solution with oxygen. Mechanical tests show a significant increase in hardness with the increase of injected n-Al_2_O_3_ amount. A maximum of 620 HV was measured that is almost 4 times higher than the pure LMD-printed Ti structure.

## 1. Introduction

Metal matrix composites with nanoparticles (NP) as reinforcement (MMnCs–metal matrix nanocomposites) have been investigated during the last decades and significant improvements in the mechanical properties such as tensile strength, elastic modulus and wear resistance were demonstrated [1,2,3]. The higher performances were explained by the cumulative contributions of different strengthening mechanisms promoted directly or indirectly by the dispersed nanoparticles. Among these mechanisms, load transfer, Hall-Petch strengthening, Orowan strengthening, coefficient of thermal expansion and elastic modulus mismatches are often mentioned [4]. Furthermore, it has been shown that the processing route may also contribute to some strengthening mechanisms through grain refinement and increased dislocation densities [5,6].

Numerous works have shown the processability of a wide range of MMnCs [1,7]. Thus, Fe, Cu, Ni, Ti or Mg matrices were reinforced with nanoparticulate materials like oxides, nitrides, carbides, boride, carbon nanotubes or graphene. Traditional fabrication routes based on powder metallurgy or casting processes have then been purposely optimized in order to avoid NP agglomerates within the metallic matrix.

The high diversity of the synthesis routes of MMnCs gives numerous possibilities to their development and production at industrial scale.

The recent emergence of Additive Manufacturing (AM) processes could further extend the range of applications of MMnCs. Indeed, the additive approach unlocks the geometric restrictions of the traditional production processes and offers more freedom in terms of material combinations. Therefore, AM opens the door to the production of unique materials accurately optimized and specifically dedicated to given applications.

Among the additive manufacturing processes for metal-based materials, the Laser Metal Deposition (LMD) seems the most promising for the fabrication of near-net shaped structures of multi-materials. LMD belongs to the generic Direct Energy Deposition (DED) processes in which feedstock, mostly in the form of a wire or powders, is directly deposited into a melt pool. The 3D structure then grows through solidification upon cooling. The energy source is generally an electron beam or a laser. More exhaustive information about the different DED processes can be notably found in References [8,9]. LMD technology enables the injection of different kinds of powders at chosen locations during material fabrication. Therefore, composites parts can be built freely within a complex-shaped matrix, bringing locally new functionalities and/or improved properties. The possibility to fabricate gradient materials, dissimilar materials as well as metal matrix composites are numerously reported in the literature. Carrol et al. [10] and Onuike et al. [11] have shown, for example, the possibility to produce functionally graded materials (FGMs) of Inconel/Stainless Steel and Inconel/Ti6Al4V, respectively. FGMs of Ti6Al4V/Mo and Ti/Nb are also reported in the recent papers of Schneider-Maunoury [12,13]. Several dissimilar material systems have also been investigated. For example, different strategies were developed for the junction of stainless steel with zirconium [14] or with titanium [15,16]. Several works on metal composites manufactured by LMD have also been carried out and reviewed among others by Bandyopadhyay et al. [17] and Fereiduni et al. [18].

However, up to now, to the best of the author’s knowledge, nobody has reported the direct injection of nanoparticles in the laser melted metal to achieve 3D structures of nanocomposite materials. All the research works on the fabrication of MMnCs with LMD or similar processes have systematically involved either premixing steps of the nano-reinforcements with the matrix microscale powder (dry or liquid processes) or have proceeded with in-situ strategies. Ball-milling [19,20,21] or the preparation of slurries that are subsequently dried [22,23] are often used to decorate the microsized matrix particles with nano or sub-nanoscale reinforcements prior printing. However, such approaches do not allow to adjust freely and locally the content of the reinforcement and consequently the properties of the printed material.

This paper summarizes the results of a preliminary study dealing with a new approach to fabricate metal matrix nanocomposites with the LMD process. Dry Al_2_O_3_ nanoparticles are directly injected into the titanium metal melt or together with the Ti micropowder in order to locally modify the mechanical behaviour. With this new processing method, neither premixing nor decoration of the microscale matrix particles is required. In addition, this new method offers better flexibility to change locally the mechanical response of the additive manufactured parts depending on the requirements. Thus, two strategies to introduce alumina nanoparticles into a titanium matrix in LMD process are discussed and the resulting microstructures and hardness are compared with pure titanium 3D structures.

## 2. Materials and Methods

### 2.1. Materials

Four millimetre Ti plates of Grade 1 supplied by Zapp AG were used as material substrate for building the investigated 3D parts. Ti Grade 1 spherical micropowder (45–105 µm) and nano-Al_2_O_3_ powder (Aeroxide Alu65, equivalent mean particle size of 23 nm) ordered from Advanced Powder and Coating (AP&C) and Evonik, respectively were used as feedstock for the different nanocomposites. According to the provider certificate, the titanium powder contains 800 ppm oxygen.

### 2.2. Fabrication of n-Al_2_O_3_/Ti Walls

A commercial LMD machine (Mobile 1.0, BeAM, France) was used for building the 3D structures in the shape of a wall (10 × 6 mm).

This LMD system comprises an airtight process chamber offering the possibility to work under a low level of oxygen and a continuous wave (CW) fibre laser with a maximum power of 500 W operating at wavelength of 1068 nm (YLR-Series, IPG Photonics). The focal spot diameter of the laser is about 800 µm. The machine is conventionally equipped with a volumetric powder feeder (Medicoat, Switzerland) with two powder containers. In addition to the feeder, a specific dense phase convey system (Impakt^TM^, Powder and Surface GmbH, Germany) has been integrated into the LMD setup. The feeder is specifically designed for the transport of non- or poorly flowable powders, like nanoparticles.

**Figure 1:**
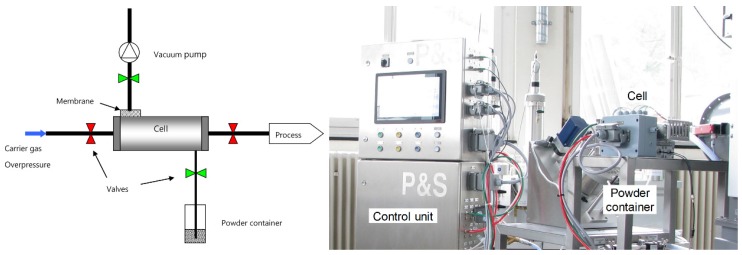
Scheme and picture of the Impakt powder feeder for non-flowable powders.

A schematic representation of the powder feeding system and its principle is depicted in Figure 1. A cell having a specific volume is equipped with four pneumatic valves, which are able to work at high frequency. First, the cell is evacuated using a vacuum pump and then the powder is sucked from the powder container under argon atmospheric pressure for a specific time. After this step, an overpressure of argon is built into the cell before opening the valve leading to the process. The floating powder is then transported by pressure difference. Using many cells in parallel and a process cycle, the powder feed may appear quasi-continuous but actually reaches a pulsed feeding regime up to 10 Hz.

Thus, the titanium microscale powder was fed with the volumetric conventional powder feeder while the alumina nanoparticles were transported using the Impakt powder feeder system. Both powders were injected coaxially to the laser spot in the process zone through a specific conical nozzle. The focus distance of the nozzle for both the laser beam and the powder jet was 3.5 mm. The head was supported by a 3 axes system (x,y,z) and the substrate was fixed on a 2 rotative axes holder. The samples were produced in argon atmosphere (15 ppm O_2_, 100 ppm H_2_O).

**Figure 2:**
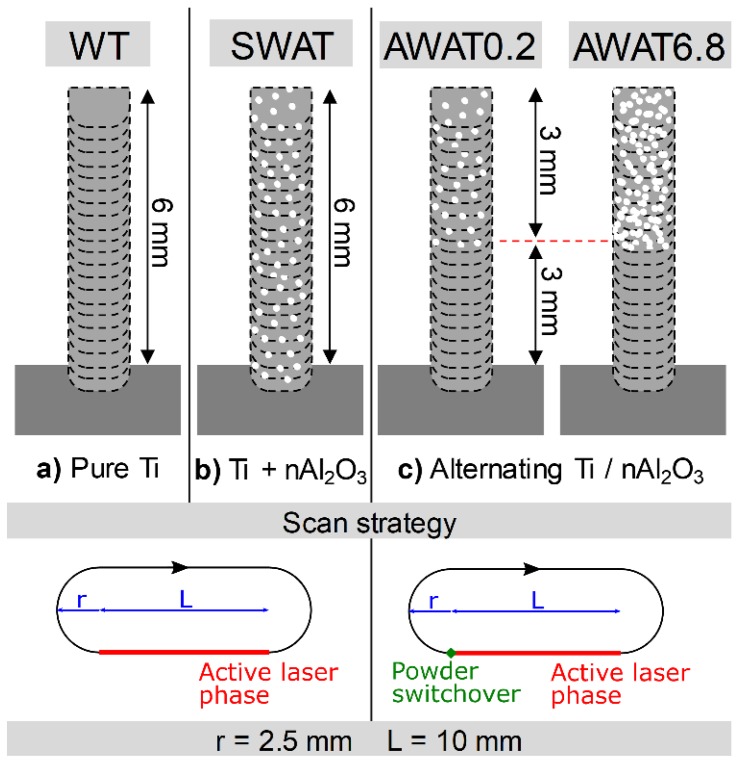
Scheme of the different produced samples. (**a**) WT; (**b**) SWAT; (**c**) AWAT.

Pure Ti walls (denoted in the following as WT) were fabricated with a laser power of 325 W and a scanning speed of 2000 mm min^−1^ with a z increment of 0.2 mm. The laser head following a stadium geometry on a plane parallel to the substrate surface (Figure 2) was chosen as the scanning strategy. The semicircles had a centre-to-centre distance of 10 mm and a radius of 2.5 mm. The laser was activated on one straight side while completing the turn in an inactive state leading to the material printing process. The speed during the inactive laser phase was set at 50 mm·min^−1^. For all the experiments, the Ti powder mass flow was kept constant at 4 g·min^−1^.

Beside pure titanium walls, three different types of samples were fabricated with nanoparticles.

For the first type, simultaneous injection of both powders was used (sample referred to in the following as SWAT). For the other two types, the injection of titanium and alumina was alternated within each layer (samples referenced as AWAT). Schematics of the different samples are depicted in Figure 2.

For the printing of the simultaneous injection of n-Al_2_O_3_ and Ti powders (SWAT), the same printing parameters were used except the laser power which was increased to 365 W. This was done in order to compensate for the shadowing of the laser beam caused by the increase in the total powder flow. The n-Al_2_O_3_ mass flow was 0.2 g min^−1^. The cycle time of the n-Al_2_O_3_ powder feeder was set to 150 ms which resulted in a powder pulse of 13.33 Hz.

Walls with an alternated injection of Ti and n-Al_2_O_3_ powders were printed with the same laser power and scanning speed as for pure Ti samples. Two different mass flows of alumina powder were used: 0.2 and 6.8 g min^−1^, the corresponding samples are denoted as AWAT0.2 and AWAT6.8, respectively. A 3 mm high wall of composite was deposited on a 3 mm wall of pure titanium (Figure 2c). The deposition was carried out with the following sequence: the n-Al_2_O_3_ and the subsequent Ti layer were printed at the same height without any z increment. The z distance was then incremented by 0.2 mm and the cycle was repeated. The switch from one to the other powder feeding system took place at the beginning of the inactive laser phase in order to guarantee that the targeted powder was injected while processing. This procedure limited also the cross-contamination of the powders and improved the stability of the powder flow.

### 2.3. Characterization Methods

Cross-sections of all the samples were prepared for analysis. The walls were therefore cut, embedded in a Struers 2K resin, ground up to grid 4000 SiC grinding paper and mirror polished with OPS solution (0.04 µm SiO_2_ mixed with H_2_O_2_). The cross-sections were examined with an optical microscope (ZEISS Axioplan, Germany) after being etched with a Kroll reagent. Crystal structures were analysed by XRD (Bruker D8 discover, Germany). The average grain size was calculated with the linear intercept method on optical micrographs.

Further investigations of grain morphology, chemical composition and elemental spatial distribution were performed using a Scanning Electron Microscope (SEM–Mira Tescan 3, Czech Republic) equipped with an energy dispersive X-ray spectrometer (EDS–Ametek Edax Octane plus, USA) and electron backscatter diffraction camera (EBSD–Ametek, USA). The EDS and EBSD maps were obtained with an acceleration voltage of 20 kV, with a 1 µm step size. Nanopowder distribution and phases within the Ti matrix were further investigated by scanning transmission electron microscopy (TEM, JEM2200-FS, Jeol, USA). The microhardness HV_0.5_ was measured with a load of 500 g and a dwell time of 10 s. A minimum of 5 indentations was used for each identified structure to determine the average hardness.

## 3. Results and Discussion

### 3.1. Pure Ti Wall (WT) Printed by LMD

**Figure 3:**
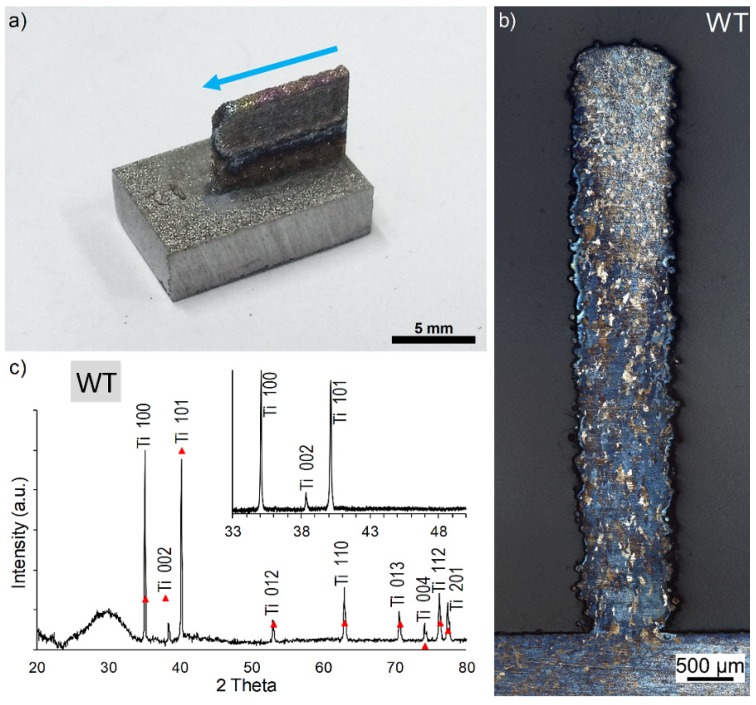
Macroscopic (**a**), microscopic (**b**) observation and X-ray diffraction (XRD) pattern (**c**) of the Ti Wall (WT) sample. The blue arrow indicates the direction of the printing.

Dimensions of the samples WT (Figure 3) are close to the programmed ones (10 × 6 mm), with a length of 9.95 mm and a height of 6.40 mm. The width is around 900 µm, which is in agreement with the laser beam diameter, even if at the interface with the substrate the 3D structures were always thinner due to enhanced heat release through the metallic substrate. No cracks can be observed and the substrate was not deformed after printing.

Optical microscopic observations of the etched cross-sections (Figure 3b) confirm that no crack occurred at the interface with the substrate and reveal columnar grains oriented in the growth direction having an average length and width of 47.8 ± 8.2 µm and 59.8 ± 13.3 µm, respectively. The preferential orientation is promoted by the temperature gradients (G) and cooling rate (R) typically observed for laser-based additive manufacturing processes [24,25].

Only peaks of the α Ti phase are identified in the XRD pattern (Figure 3c). One can note the higher intensity of the (100) plane when compared to the standard α Ti phase because of the specific orientation of the grains. The large round peak observed around 30° is caused by the polymer matrix in which the sample was embedded.

An average hardness value of 162 ± 25 HV_0.5_ for the WT sample is measured all over the structure. This value is in agreement with the hardness values already reported for pure titanium deposited by additive manufacturing processes [12,26].

### 3.2. n-Al_2_O_3_/Ti Wall Printed with Simultaneous Injection (SWAT)

**Figure 4:**
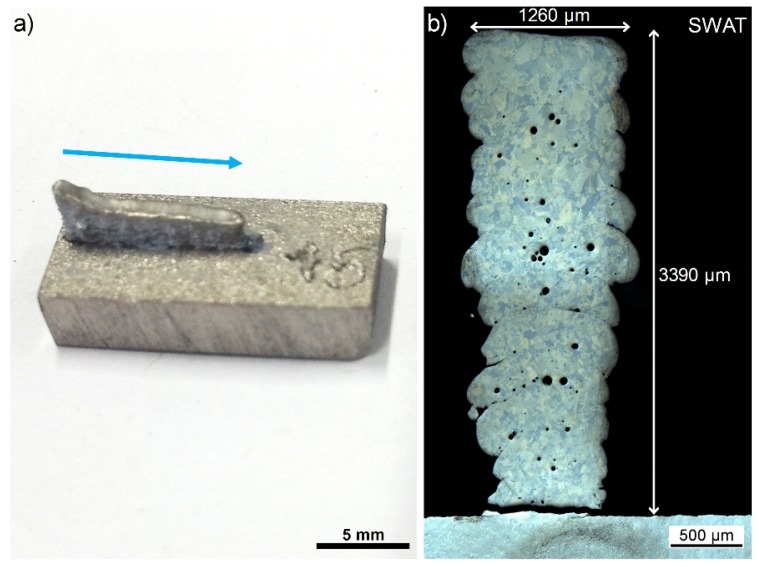
Macroscopic (**a**) and microscopic (**b**) observation of the SWAT sample. The blue arrow indicates the direction of the printing.

The SWAT sample shows a length close to the WT sample (9.38 mm). However, the height is lower than 6 mm and is presenting a curved profile. A maximum value of 3.40 mm and a minimum of 2.90 mm are measured (Figure 4a). This curved profile was also noted in previous samples (not presented in this paper) obtained with non-optimized printing parameters. First investigations have shown that this was due to an uncontrolled decrease in the titanium powder flow caused by the injection of n-Al_2_O_3_ powders during printing. Further process optimization is required to prevent the disturbance of the Ti powder flow.

The wall was well attached to the substrate that did not deform. No cracks could be seen on the as-printed part (Figure 4a). However, the cross-section reveals a large crack at the interface with the substrate (Figure 4b). This may have been induced during the preparation of the sections but could indicate a larger stress level as for pure titanium samples.

The width of the SWAT sample presents a “V” shape increasing from 870 µm at the basis to 1260 µm at the top (Figure 4b). The width at the basis is in the same range as the one measured over all the different pure Ti samples. The cross-section also clearly shows a layered structure having a layer thickness at 185 ± 9 µm in agreement with the z increment of 200 µm. Round pores are observed at the interfaces between two layers and also within layers. These pores generally assigned to gas trapped in the melt can be explained by the higher gas flow needed for the simultaneous injection of the two powders. Indeed, for avoiding rapid clogging of the nozzle, the carrier gas flow for the nanoparticles was increased to over 20 l min^−1^.

Concentrations of 4.74 at% and 13.5 at% for aluminium and oxygen, respectively are measured by EDS in the layers. The oxygen content of the pure Ti substrate below the wall is measured to be 9.7 at% (instead of 0.5 at% given by the specifications) which shows that the whole surface of the sample was contaminated, probably during the polishing step. The oxygen values are therefore not relevant and only the calculated concentration values of O in at% (assuming that alumina powder is the only source of oxygen) and the atomic ratio Al/Ti are considered for comparative analysis.

The atomic Al/Ti ratio is higher (5.80%) than the one related to the injected powders (4.70%). This difference could be induced by the powder flow fluctuations observed and mentioned previously.

**Figure 5:**
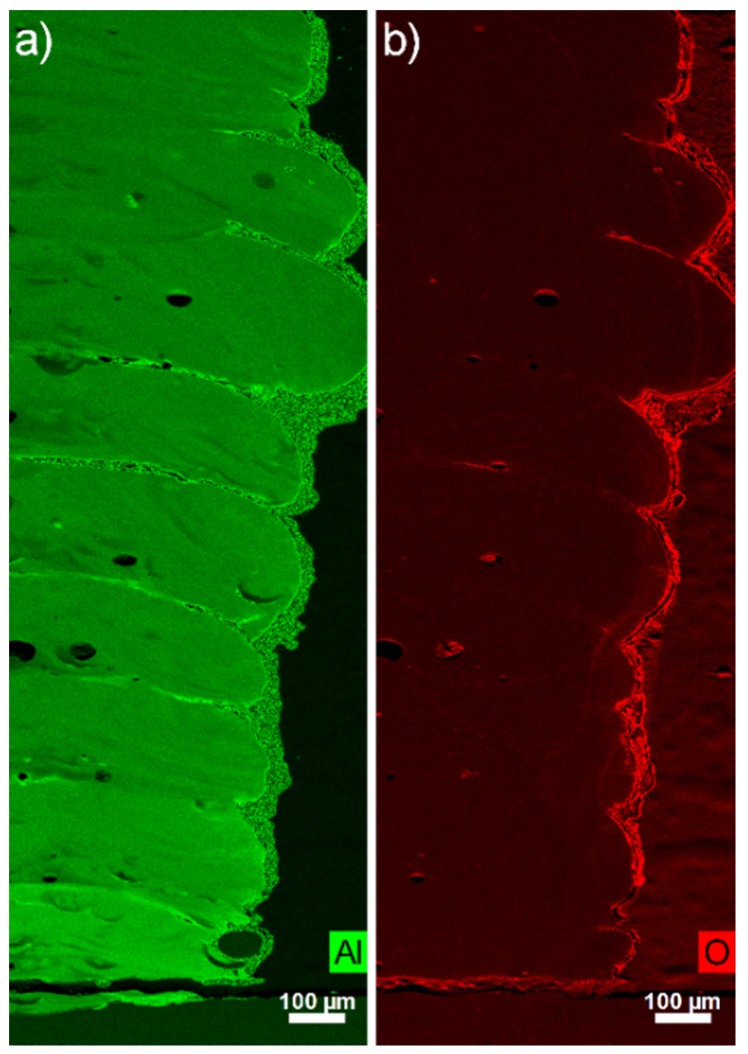
Energy dispersive X-ray spectroscopy (EDS) map measurements ((**a**) Aluminium: green, (**b**) Oxygen: red) of the sample SWAT.

Higher signal intensity for the aluminium content is measured by EDS between the layers (Figure 5a). Verezub et al. underlined in their works [27] the difficulty to incorporate nanoparticles into molten metals because of the high surface tension from the liquid in regard to the gravitational force applied on nanoparticles and their low kinetic energy. Therefore, a part of the nanoparticles could float at the surface of the molten metal during the fabrication of the wall and could remain at this location during solidification. The oxygen level seems to be only higher at the external surface of the built part and at the position of the pores (Figure 5b) and no direct correlation with the aluminium EDS signal is observed.

**Figure 6:**
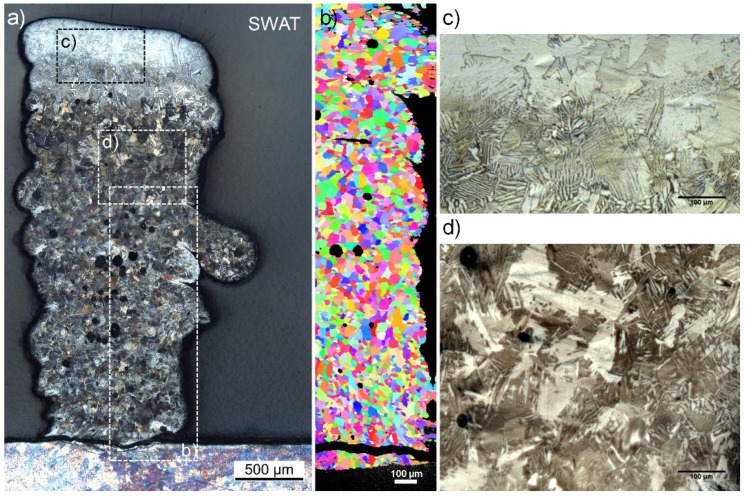
Microscopic observation (**a**), Electron backscatter diffraction (EBSD) map (**b**) and detail of the microstructure (**c**,**d**) of the SWAT sample.

According to the EDS measurements, the last 600 µm of the top of the wall (not visible in Figure 5—see the white area in Figure 6a) attests to a significantly higher Al/Ti ratio (6.33%). The aforementioned assumption used to justify the curved profile of the sample can also explain the large increase of Al content at the top part which can be caused by a sudden uncontrolled reduction of the Ti powder flow rate.

The alumina addition has a noteworthy influence on the microstructure compared to the columnar grain of WT. A homogeneous structure of fine lamellar grains (39.5 ± 2.6 µm) can indeed be identified in Figure 6a,d. This lamellar structure can however not be detected by EBSD, probably because of the too low-resolution of the scan (Figure 6b).

A microstructure composed of fine lamellar grains (41.7 ± 3.4 µm) and large platelets (165.4 ± 47.3 µm) is also clearly distinguished for the last 600 µm at the top of the wall where a higher Al content is measured (Figure 6c).

**Figure 7:**
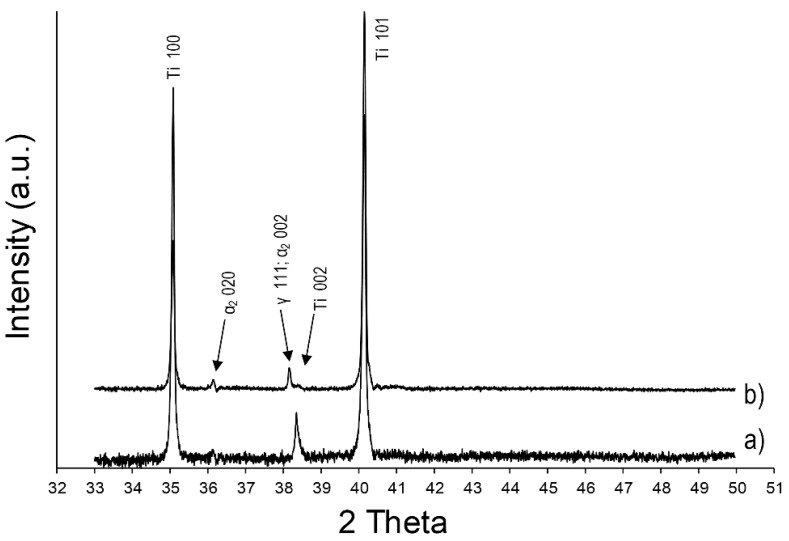
XRD patterns of the WT (**a**) and SWAT (**b**) samples.

XRD analyses show different patterns for the pure titanium samples and the parts produced with the co-injection of alumina nanoparticles. In addition to the α Ti peaks, two others can be observed at 36.1° and 38.2° for the SWAT sample (Figure 7). According to the literature [28,29,30], these peaks can respectively be associated with the presence of α_2_ (Ti_3_Al) and γ phase (TiAl) of the titanium aluminide intermetallics.

The solubility limit of Al into Ti is reached for an atomic ratio Al/Ti around 11% [31]. However, oxygen (calculated at 7.11 at% for this sample) decreases the solubility limit and induces the precipitation of the α_2_ phase for lower Al content [31,32]. This could explain the presence of Ti_3_Al and TiAl phases in the sample SWAT (Al/Ti ratio of 5.80%).

The lamellar structure is typically ascribed in the literature to the formation of plates of α_2_ phase being separated by the γ phase [33]. However, due to the relatively high average amount of oxygen (calculated at 7.11 at%) in this sample, it could be reasonable to assume that a part of the lamellar grains is formed of α_2_ plates being separated by Ti in a solid solution with oxygen. Indeed, the solubility of oxygen in Ti is known to be high (around 34 at%) [34] and no peaks related to the titanium oxide phases were observed in the XRD pattern (Figure 7).

The brittleness of the intermetallic α_2_ phase of titanium [33] could explain the large crack observed at the interface between the basis of the wall and the substrate. The high cooling rates (10^3^ to 10^4^ Ks^−1^) reported in the literature [9,35] for LMD processes are known to generate residual stresses which can be less accommodated by this brittle phase.

A homogeneous hardness (without considering the 600 µm top area) is observed all over the SWAT sample. When compared to the pure Ti printed wall, a higher average value of 511 ± 28 HV_0.5_ is obtained. This can be explained by the presence of Ti_3_Al phase and lamellar structures are well known to increase the strength of Ti-Al materials [29,33].

The measured values are also significantly higher when compared to titanium aluminide having a similar microstructure (fully lamellar structure) or having a similar Al/Ti atomic ratio. Tlotleng [36] reported a hardness of 400 HV_0.5_ and Qu et al. [37] mentioned a hardness ranging from 300 to 380 HV_0.2_ for fully lamella α_2_ /γ microstructures printed by LMD. Kornilov et al. [38] measured hardness values of 176 HV_10_ for a sample having an Al/Ti atomic ratio of 5.3%.

The α_2_/γ phase ratio plays an important role in the strength of the titanium aluminide intermetallic materials as reported by Kornilov et al. [38] and can justify the higher hardness of the SWAT sample when compared to the samples produced by Tlotleng [36] and Qu et al. [37] which contain a much higher Al/Ti ratio (31.7% and 94%, respectively). Moreover, the oxygen solid solution in titanium is well known as strengthening mechanism [31,34].

**Figure 8:**
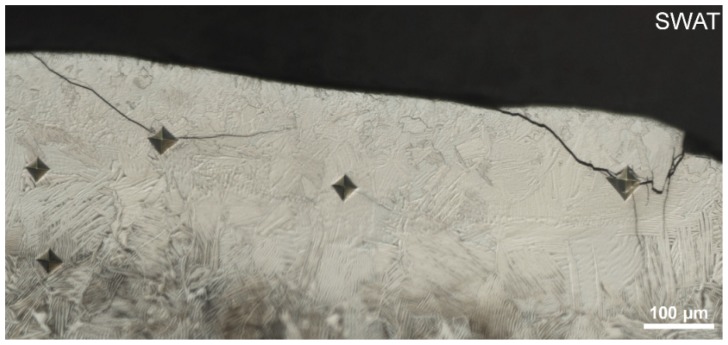
Indentation prints in the 600 µm top area of the SWAT.

The hardness measured in the 600 µm top area is even higher (588 ± 39 HV_0.5_) and cracks induced by the indentation are observed (Figure 8). An increase in the α_2_ brittle phase content could cause a decrease in fracture toughness.

The composite microstructure constituted of fine Ti_3_Al lamellas within a Titanium strengthened with an oxygen solid solution (with the possible presence of Ti_3_Al/TiAl lamellar grains) shown by the SWAT sample is therefore beneficial for the hardness improvement.

### 3.3. n-Al_2_O_3_/Ti Wall Printed With Alternated Injection (AWAT)

**Figure 9:**
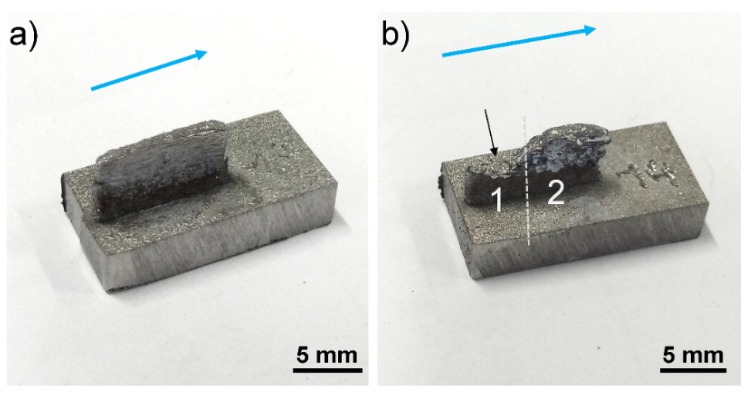
Macroscopic observations of the AWAT0.2 (**a**) and AWAT6.8 (**b**) samples. The blue arrow indicates the direction of the printing. The black arrow shows the rough surface of part 1 of AWAT6.8.

**Figure 10:**
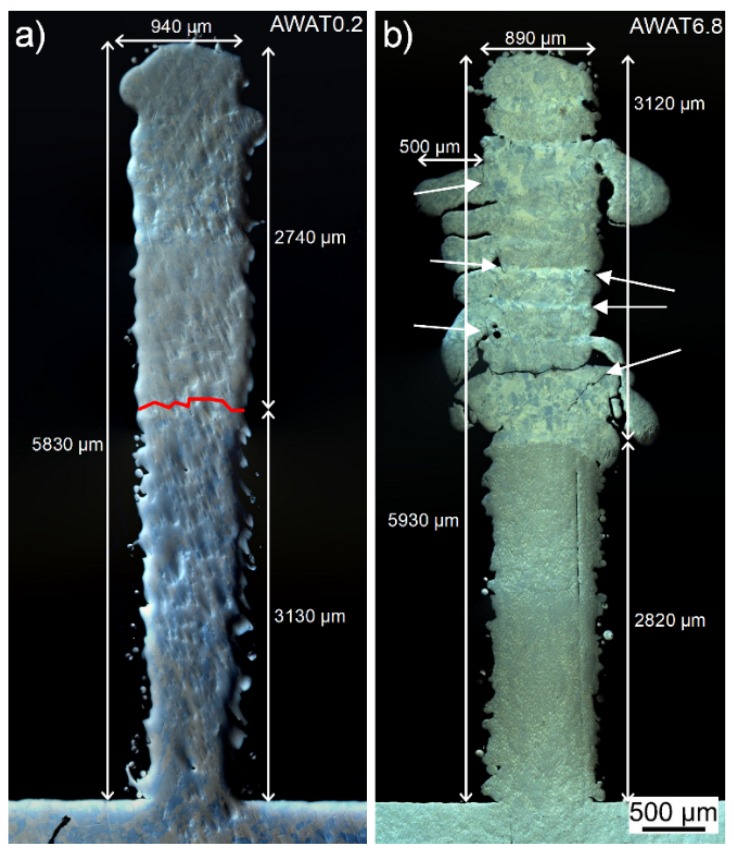
Microscopic observations of the AWAT0.2 (**a**) and AWAT6.8 (**b**) cross-sections. The white arrows indicate cracks.

The AWAT0.2 sample (Figure 9a and Figure 10a) obtained with a low mass flow of nano-alumina shows regular dimensions close to the reference sample with a 10.02 mm length and a 0.94 mm width. A lower height (5.83 mm) than the expected value (6 mm) is measured. Neither cracks nor pores are observed.

The AWAT6.8 sample with higher nano-alumina content shows on the contrary several cracks located between the layers. The height is uneven and two main different values can be measured (Figure 9b). The lowest part (part 1 in Figure 9b) has a height reaching a maximum of 3.50 mm while the second part (part 2) is slightly lower than the expected value (5.93 mm). This height difference could be explained by the delamination during the printing process of the upper part of the wall (formed by the alternation of nano-Al_2_O_3_ and Ti). The rough surface observed at the top of part 1 of the wall (see black arrow in Figure 9b) as well as the presence of cracks visible in part 2 of the wall (white arrows in Figure 10b) strongly support this hypothesis.

Although the width (890 µm) of the lower part of the wall (pure Ti) of AWAT6.8 and its length (9.32 mm) are similar to the dimensions of the WT sample, the width of the upper part is highly uneven due to layers partly spread out of the wall (Figure 10b). The thinnest width of this upper part is measured at 875 µm while the largest is measured at 1390 µm.

**Figure 11:**
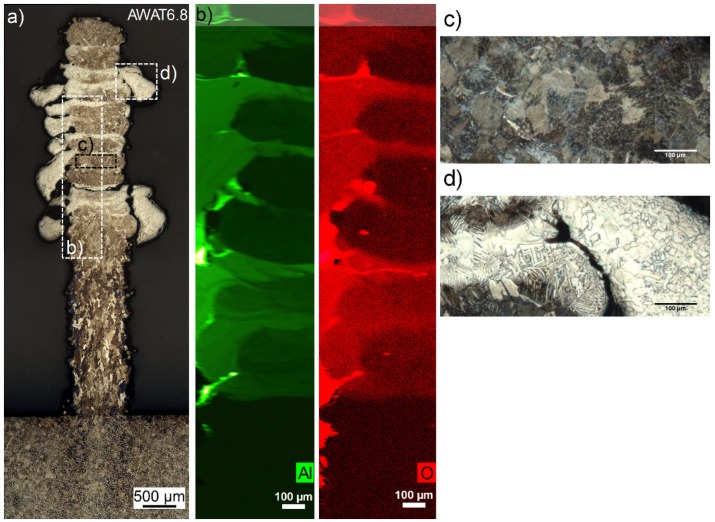
Microscopic observations (**a**), EDS maps (Aluminium: green, Oxygen: red) (**b**) and detail of the microstructure (**c**,**d**) of the AWAT6.8 sample.

For the sample AWAT6.8, distinctive alteration of brown and beige layers are observed for the etched cross-section (Figure 11a). The brown layers (thickness 200.8 ± 47.5 µm) constitutes of lamellar colonies and equiaxed grains (38.6 ± 5.5 µm) as seen in Figure 11c, whereas the beige ones (thickness 60.9 ± 12.1 µm) are formed by a mixture of large lamellar grains and coarse platelets (58.8 ± 11.1 µm) (Figure 11d). According to EDS measurement, the brown and beige layers have atomic Al/Ti ratios of 4.58% and 12.76%, respectively. EDS mapping of O shows that the areas with the highest content of O correspond to the Al-rich areas (Figure 11b). According to these observations, the brown and beige layers should correspond to the injection of pure titanium powder and injection of pure n-Al_2_O_3,_ respectively.

The high Al/Ti ratio in the brown layers can be caused by the diffusion of Aluminium originally located in the Al-rich layers toward these layers which could be promoted by the partial remelting and reheating generated by the repeated back-and-forth scanning movement of the laser during the whole fabrication of the wall structure. Powder cross-contamination during the alternation of the injected powder (from n-Al_2_O_3_ to Ti powders) could also explain the presence of Al in the brown layers.

Based on the EDS maps (Figure 11b), the average thickness of the aluminium-low and oxygen-low layer is calculated at 190.1 ± 32.3 µm. Without considering the spread parts, the thickness of the Al/O-rich areas measured in the longitudinal middle part of the wall is 62.4 ± 15.2 µm. These values are in agreement with the thickness measurements obtained from the etched cross-section (Figure 11a).

The spreading of the Al/O rich layers could be due to the difference in reflectance of Ti (0.61 [39]) and Al_2_O_3_ (0.07 [40]) at the 1068 nm wavelength. Consequently, for the same laser power, the energy absorbed by Al_2_O_3_ is about ten times higher than that for Ti. In addition, the roughness of an irradiated surface is also improving the coupling efficiency of a laser beam. It is indeed well established that rough surfaces have higher laser absorptivity than polished surfaces. In the present study, this roughness can be increased by melt pool instabilities enhanced by the pulsed regime of the nanoparticle feeding system but also by the presence of nanoparticles at the surface of the melt pool. Zhou et al. reported that nanopowders floating at the surface of a melt pool result in multiple absorptions of the laser beam [41]. Even if probably a relative thick layer of nanoparticles is required for multiple absorption, laser scattering could already occur with a thin layer or even isolated particle agglomerates. Therefore, it is suggested that a higher energy is absorbed in the melt pool when nanoparticles are directly injected decreasing the viscosity of the Al/O rich layers. In order to compensate for this increased absorption, a reduction of the laser power is considered for future experiments.

Further investigations should provide more data to explain the fact that the highest height (part 2 in Figure 9b) of AWAT6.8 is close to 6 mm despite the spreading of the Al/O-rich beige layers. Indeed, the thickness of the Al-poor layers measured for this sample is significantly high and the total thickness (addition of the average thickness of the brown + beige layer) exceeds the z increment.

**Figure 12:**
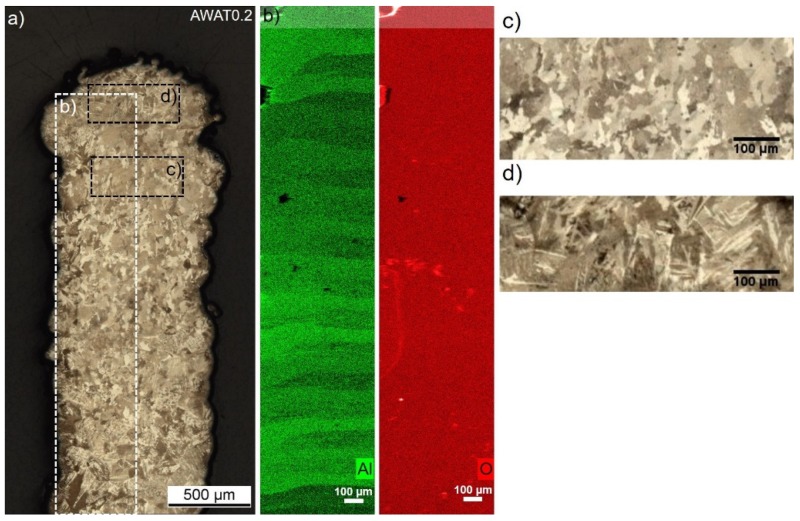
Microscopic observations (**a**), EDS maps (Aluminium: green, Oxygen: red) and (**b**) detail of the microstructure (**c**,**d**) of the AWAT0.2 sample.

The alternation for the sample AWAT0.2 of low Al/Ti atomic ratio layers (0.97%) made out of fine columnar grains (23.8 ± 0.8 µm) and richer Al layers (Al/Ti ratio at 1.55%) formed by lamellar grains (62.3 ± 7.7 µm) can also be observed (Figure 12c,d respectively). Oxygen seems to be homogeneously distributed all over the sample (Figure 12b).

The presence of a few atomic percent of Al into the low Al layers could explain the size refinement of the columnar grains when compared to the pure Ti sample (WT). Impurities in a liquid can indeed promote heterogeneous nucleation inducing a refinement of the grain dimension.

According to the EDS mapping of aluminium (Figure 12b), the rich Al areas show an even thickness being 62.8 ± 18.2 µm, whereas the layers having a lower Al content were measured to have a thickness of 114.9 ± 14.3 µm. The addition of the low and rich layers average thickness (total thickness) is close to the z increment.

**Figure 13:**
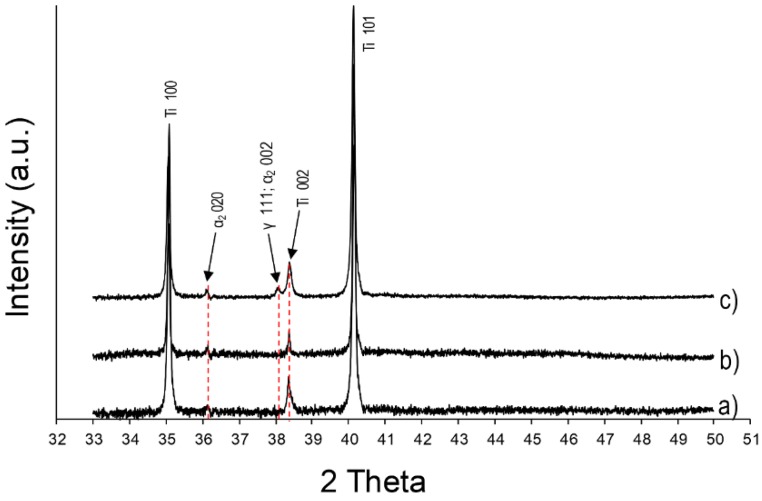
XRD patterns of the WT (**a**), AWAT0.2 (**b**) and AWAT6.8 (**c**) samples.

As already described for the sample SWAT, the lamellar structure observed in the layers of AWAT0.2 and AWAT6.8 containing aluminium can be identified as the α_2_ phase (Ti_3_Al). XRD pattern of the sample AWAT6.8 (Figure 13c) shows distinct peaks of this phase. The presence of TiAl (γ phase) into this sample can also be assumed because of the appearance of the peak located at 38.2°.

Despite the observation of lamellar colonies within the Al-rich layers of AWAT0.2, Ti-Al intermetallic phases are more difficult to be identified by XRD. Due to the noise of the measurement, the apparent peak at 36.1° (Figure 13b) cannot be objectively be ascribed to the α_2_ phase. Neither peaks of Al_2_O_3_ nor peaks of TiO_2_ could be detected.

Moreover, no clear evidence of the presence of nanoparticles within the metallic matrix of the AWAT0.2 sample could be detected from the HR-TEM investigations. HR-TEM analysis of AWAT6.8 and SWAT samples is still ongoing.

Considering that no residual n-Al_2_O_3_ particles could be detected even with TEM analysis and that both Ti_3_Al and TiAl (for the SWAT and AWAT6.8 samples) phases were detected, it can be assumed that the nanoparticles are partially or totally dissolved within the Ti melted pool. Aluminium as well as oxygen are promoting the formation of titanium aluminide phases and additionally oxygen is contributing to the strengthening of the Ti matrix through a solid solution.

Neither dissolution of Al_2_O_3_ nor apparition of titanium aluminide phases was noted by Zhang et al. [42] in the LMD-printed structures with microscale Al_2_O_3_ and Ti powders. They reported unmelted Al_2_O_3_ particles in an inhomogeneous microstructure of mixed Ti and Al_2_O_3_ phases. The nanosize factor could therefore enhance the dissolution of the Al_2_O_3_ particles and increase the reactivity with Ti.

**Figure 14:**
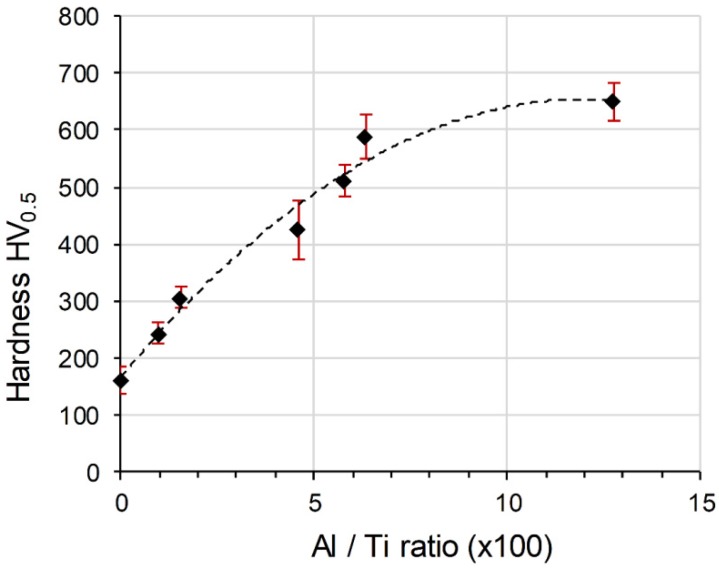
Evolution of the hardness as a function of the atomic Al/Ti ratio.

The hardness evolution of the two AWAT samples (306 ± 18 HV_0.5_ and 244 ± 19 HV_0.5,_ respectively for the rich and poor Al phases of AWAT0.2 and 620 ± 33 HV_0.5_ and 425 ± 52 HV_0.5_ for AWAT6.8) demonstrate the strong correlation between the Al and O content with the hardness. Indeed, the increase in the Al/O content within the Ti matrix can clearly be related to the increase of hardness as shown in Figure 14. The decrease of the slope for higher Al/Ti ratio could be explained by the increase in the α_2_/γ ratio which induces the reduction of the fine lamellas and the appearance of bigger platelets structure.

As hypothesized for the SWAT sample, the significant hardness improvement for the two AWAT samples can be attributed to the presence of the hard α_2_ lamellar phase and to the strengthening effect of the oxygen solid solution in the Ti matrix. The increase in hardness of the Al-low layers in AWAT0.2 when compared to the WT despite the absence of the lamella microstructure can be explained by the strengthening effect of the O solid solution in Ti as well as probably the grain refinement (Hall-Petch effect). These results show the possibility to improve the mechanical performances of a 3D printed structure without the requirement of premixing or blending steps.

Further mechanical investigations of the composite microstructure constituting fine α_2_ lamellar phase within a Ti matrix being strengthened with the O solid solution could show interesting properties. Kothari et al. [33] underlined indeed the important role of the α_2_ lamella grain and its size for the improvement of ductility, strength, fracture toughness and creep resistance. According to this review, the fully lamellar structure presents the best performance for the fracture toughness and its decreasing grain size is expected to increase the ductility and strength. Moreover, titanium with an oxygen solid solution has shown to improve mechanical properties such as strength and ductility [31]. Therefore, materials similar to the SWAT sample could show promising perspectives.

## 4. Conclusions

This work shows the capability of the direct nanoparticle injection approach to overcome the restrictions caused by the current premixing techniques or in-situ strategies used so far to manufacture MMnCs by LMD. The potential of this approach was illustrated here by producing Ti composites in which the content and the location of nano-alumina was freely adjusted during the LMD process. Therewith, it is now possible to improve the mechanical performances of the 3D structures only where needed, meaning where the external stress would be higher and no premixing or blending of the metal matrix powder with the nanoparticulate reinforcement material is required.

It has been demonstrated that the introduction of the n-Al_2_O_3_ particles into the Ti melt pool has a significant effect on the final microstructure. A α_2_ lamellar structure within a Ti matrix with oxygen solid solution is promoted leading to increased hardness. A maximum hardness around 620 HV_0.5_ has been measured for the highest content of injected alumina nanoparticles. However, no nanoparticles could be observed as they could be partially or even completely dissolved in the titanium matrix.

A spreading of the Al/O rich layers occurs for the highest nano-alumina powder flow leading to undesired geometries of the produced shape. This spreading could be due to a higher energy input which decreases the layer viscosity. This higher energy coupling efficiency can be induced by alumina that has a 10 times higher absorptivity than titanium, by the roughness of the melt pool enhanced due to high and pulsed carrier gas flow rates and also by scattering effects induced by the nanoparticles at the surface of the melt pool. Therefore, a decrease of the laser power is required for future experiments and this power reduction could also limit the dissolution of the nano-alumina within the Ti matrix.

Moreover, a few technical issues have to be further investigated to guarantee a constant and stable feeding of the nanoparticles and the metal matrix powder. This may require the development of a new specific nozzle beside the optimization of the processing parameters.
